# Comparison of two methods of collecting healthcare usage data in chiropractic clinics: patient-report versus documentation in patient files

**DOI:** 10.1186/s12998-014-0032-9

**Published:** 2014-10-01

**Authors:** Taco Houweling, Jennifer Bolton, David Newell

**Affiliations:** University Hospital Balgrist, Forchstrasse 340, 8008 Zürich, Switzerland; Anglo-European College of Chiropractic, 13-15 Parkwood Road, Bournemouth, BH5 2DF UK

**Keywords:** Patient care, Health services, Health care costs, Cost measures, Chiropractic, Complementary therapies, Questionnaires, Data collection, Clinic visits

## Abstract

**Background:**

The use of patient-reported questionnaires to collect information on costs associated with routine healthcare services, such as chiropractic, represents a less labour intensive alternative to retrieving these data from patient files. The aim of this paper was to compare patient-report versus patient files for the collection of data describing healthcare usage in chiropractic clinics.

**Methods:**

As part of a prospective single cohort multi-centre study, data on the number of visits made to chiropractic clinics determined using patient-reported questionnaires or as recorded in patient files were compared three months following the start of treatment. These data were analysed for agreement using the Intraclass Correlation Coefficient (ICC) and the 95% Limits of Agreement.

**Results:**

Eighty-nine patients that had undergone chiropractic care were included in the present study. The two methods yielded an ICC of 0.83 (95% CI = 0.75 to 0.88). However, there was a significant difference between the data collection methods, with an average of 0.6 (95% CI = 0.25 to 1.01) additional visits reported in patient files. The 95% Limits of Agreement ranged from 3 fewer visits to 4 additional visits in patient files relative to the number of visits recalled by patients.

**Conclusion:**

There was some discrepancy between the number of visits made to the clinic recalled by patients compared to the number recorded in patient files. This should be taken into account in future evaluations of costs of treatments.

## Background

In a climate of increasing healthcare costs and limited budgets, calculating the costs of caring for patients constitutes an essential component of healthcare evaluations. This information can assist patients and clinicians in making treatment-related decisions as well as policy-makers in allocating resources [1]. Two types of data are needed in order to calculate the cost of an intervention. Firstly, the cost of one unit of each resource (i.e. unit cost), which can be obtained from national cost tariffs, and secondly, the amount of resources used to provide that care. Resource usage data should be collected using validated measurement methods so as to produce robust study findings.

There are different methods of collecting resource usage data including patient-reported questionnaires and diaries as well as patient records [2]. A drawback of obtaining this information from patient records is the difficulty in accessing these files [3]. Patients may attend a number of different health care services necessitating accessing multiple patient records across different health care providers. Moreover, information such as time off work and over the counter medication cannot be determined using this method [4]. While cost data can be comprehensively assessed using patient-reported diaries, the amount of patient co-operation needed for their completion is a concern [2].

Patient-reported questionnaires offer an interesting alternative in assessing resource usage. This method of assessing costs allows determination of a broad range of economic data including lost productivity and out-of-pocket expenses with less effort and resources compared to accessing patient records [4]. However, the accuracy of this type of information relies on the memory of patients and thus may be affected by recall bias. There is evidence that poor health status or declining cognitive function in old age may influence the ability to recall healthcare usage [5,6]. Another factor that may contribute to inaccurate recall is the so called telescoping phenomenon in which study participants recall a single or multiple consultations that took place outside the time frame of the study [7].

Studies that have assessed the agreement between resource usage data collected from patient records and from patient-reported questionnaires have shown conflicting results [2-8]. Some authors reported good agreement and others reported considerable differences between the data collected using these two methods. A possible explanation for this controversy may be that the ability to recall resource usage is design- and population-specific [5,6,8]. For example, recall bias may be higher in elderly patient populations, or for longer recall periods.

In this context, a study assessing the accuracy of patient-reported number of visits in chiropractic clinics has not yet been conducted. The objectives of this study therefore were: i) to determine the accuracy of patient-reported number of chiropractic visits when compared to the number of visits recorded in patient files, and ii) to determine whether the accuracy of patient-reported visits is dependent on the frequency of visits.

## Methods

### Study sample

The present study was part of a prospective single cohort multi-centre study in which outcomes, patient experiences and related costs of care were documented in low back pain patients undergoing chiropractic treatment (Houweling T: Description of outcomes, patient experiences and related costs of care in low back pain patients undergoing chiropractic treatment in the UK, unpublished). Participants were recruited consecutively between August 2010 and July 2011 from chiropractic clinics in the United Kingdom, and were assessed prior to the initial consultation in clinics and at three months follow-up by mail using questionnaires. All members of the British Chiropractic Association were invited to participate in the study by collecting data from a maximum of 10 eligible patients each. Inclusion criteria were low back pain patients over the age of 18 with or without leg pain who had not received any treatment for their condition, apart from their GP, in the previous three months*.* All participants gave written consent to participate, and ethics approval was granted by the Anglo-European College of Chiropractic Research Ethics Sub-Committee.

### Variables of interest

Patient clinical and socio-demographic characteristics, including health status, were assessed in the baseline questionnaire. Health status was depicted by the Bournemouth Questionnaire, a validated multidimensional outcome measure for use in low back and neck pain patients [9,10]. Participants were asked in the follow-up questionnaire, amongst other information, how many visits they had made to the chiropractic clinic for low back pain since they completed the baseline questionnaire. The number of visits as reported in patient files was determined in 17 clinics that were randomly selected from the participating clinics database using computer generated numbers. These clinics were sent a form requesting the number of visits participants made to the clinic between baseline and follow-up data collection points as recorded in the patient’s file.

### Data analysis

Patient characteristics of the validation sample and the complete cohort were compared using the independent t-test for continuous variables and the chi^2^ test for categorical variables. The degree of agreement between the number of visits measured using patient-report and from patient files was assessed using the Intraclass Correlation Coefficient (ICC). The ICC indicates the concordance between two measures and is expressed in a score ranging from 0 (no agreement) to 1 (perfect agreement). A single measures two-way mixed consistency model (ICC_2,1_) was selected as the participant effect was random and the measure effect fixed. A drawback of the ICC is that it is dependent on sample heterogeneity, hence this calculation was complemented by the evaluation for comparing methods by Bland and Altman, which indicates a range of differences between methods to be expected for any individual participant [11]. This approach involved plotting the differences between patient-reported visits and visits as based on patient files (patient file – patient-report) against their mean as well as calculating the 95% limits of agreement. The significance of the relationship between the differences versus the mean was assessed using Spearman’s correlation coefficient. Normalisation of the data on number of visits using log-transformations had little impact on this relationship; hence the simpler analyses were presented. A sample size of approximately 85 subjects with two measures (i.e. patient-report and patient-file) each yielded a power of 92%, assuming an ICC for the alternative and null hypothesis of 0.90 and 0.80, respectively, and a two-sided significance level of 5%. All analyses were conducted using SPSS v20.

## Results

**Figure 1:**
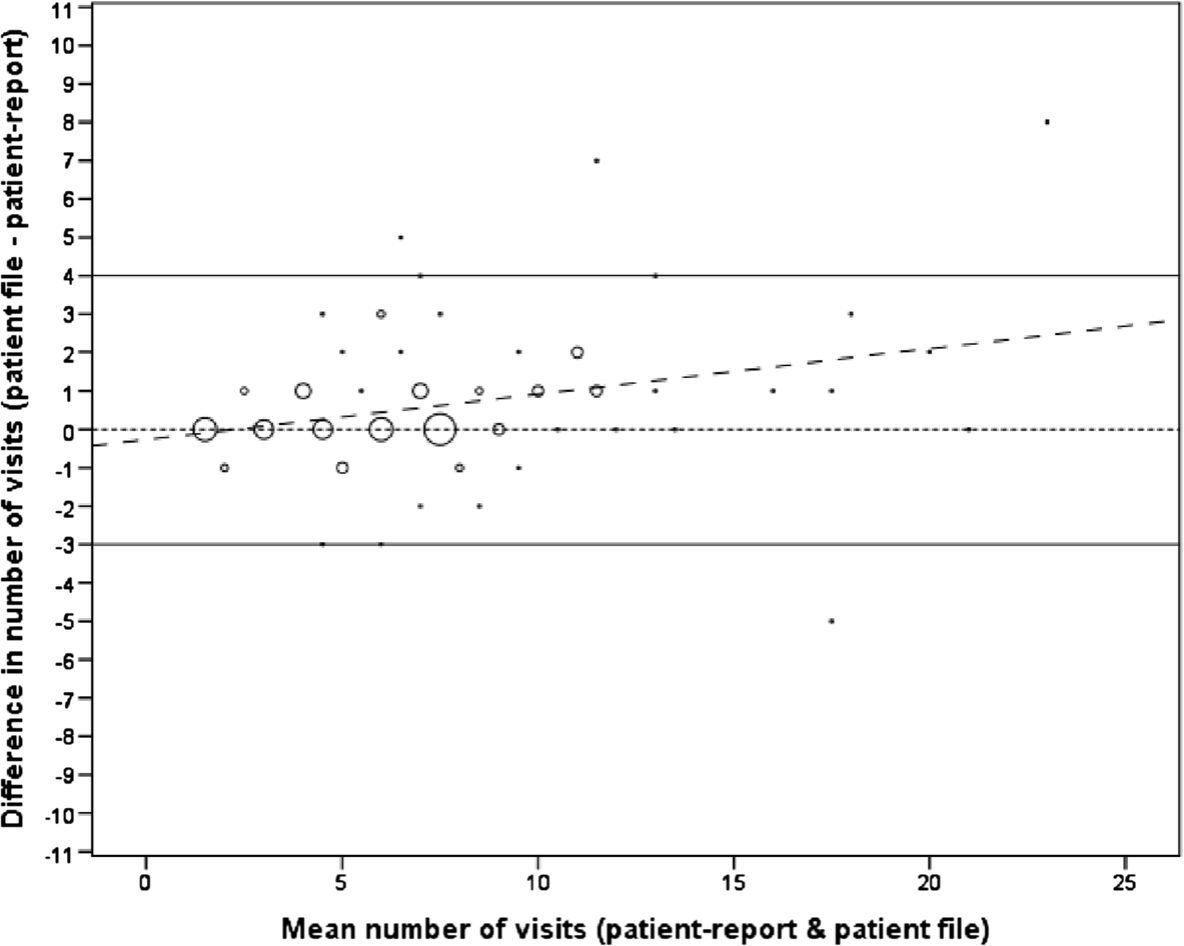
**Bland and Altman plot comparing the number of chiropractic visits as determined by patient-report and patient file.** The solid lines indicate the 95% limits of agreement, the wider dashed line represents the regression line, and the dotted line is drawn at zero (exact agreement). The size of each bubble is proportional to the number of patients with the corresponding x and y values.

Data on number of visits were available for both methods of collecting data on 89 (37%) of the 238 patients of the complete cohort, and no significant differences (p < 0.05) were found between the validation sample and the complete cohort in the distribution of clinical and demographic variables (Table 1). The ICC was 0.83 (95% CI = 0.75 to 0.88) indicating reasonable agreement between the number of visits determined using patient-report and that recorded in patient files. However, since the ICC is a relative measure, it should be viewed in light of alternative methods of determining agreement. The mean number of visits reported by patients and that reported in patient files was 4.6 (SD = 2.91) and 5.3 (SD = 3.26) respectively. Under-reporting by patients was seen in 39 patients (44%) and over-reporting in only 13 patients (15%) with a mean of 0.6 (95% CI = 0.25 to 1.01) of additional visits recorded in patient files. The Bland and Altman plot (Figure 1) illustrates the finding that patients tended to under-report visits. As might be expected, there was an increase in the variability with an increasing number of visits, with a significant upward bias indicating a trend towards under-reporting with increasing number of chiropractic visits in the previous three months (Spearman correlation coefficient = 0.295, p < 0.01). The 95% limits of agreement, a range in which 95% of the differences between methods should lie, stretched from 3 fewer visits to 4 additional visits recorded in patient files.

**Table 1:** **Baseline characteristics of the complete cohort and the validation sample**

**Variable**		**Follow-up cohort (n = 238)**	**Sample cohort (n = 89)**	*p-value
Age	Mean (SD, range) number of years	47.3 (14.45, 19–88)	46.3 (14.46, 19–88)	0.587
	Missing	2	1	
Sex	Male	104 (44)	40 (45)	0.840
	Female	134 (56)	49 (55)	
	Missing	0	0	
Work status	In paid (including self) employment	183 (77)	71 (80)	0.824
	At home and not looking for work	8 (3)	2 (2)	
	Unemployed because of back pain	1 (<1)	0	
	Unemployed because of other reasons	7 (3)	3 (3)	
	Retired	35 (15)	10 (11)	
	Student	4 (2)	3 (3)	
	Missing	0	0	
Pain history	< 3 months	84 (35)	29 (33)	0.966
	≥ 3 months	154 (65)	59 (66)	
	Missing	1 (<1)	1 (<1)	
Medication usage	Never	50 (21)	23 (26)	0.596
	Rarely	53 (22)	23 (26)	
	Sometimes	83 (35)	26 (29)	
	Every day	52 (22)	17 (19)	
	Missing	0	0	
BQ	Mean (SD) score	29.4 (15.41)	29.2 (15.65)	0.956
	Missing	5	2	

Values are frequency (%) unless stated otherwise. N = number of observations. BQ = Bournemouth Questionnaire. *Statistical significance was determined using chi^2^ test for categorical and independent t-test for continuous variables.

## Discussion

This study assessed the accuracy of patient-reported healthcare usage in chiropractic clinics at three months following the start of treatment. The results suggested that there were differences in the number of visits made to the clinic as reported by patients and the same information recorded in patient files. While these findings were in contrast to those found in two studies comparing the number of visits by patient-report and patient files in general practitioner clinics [2,3], they were similar to a study comparing both methods of collecting data in physiotherapy clinics [4]. This controversy may be explained by the fact that patients who participated in the present and in the physiotherapy research attended the clinic more frequently than those who were included in the general practitioner studies, hence making it more difficult for patients to recall the exact number of visits made. This hypothesis was supported in the present study as it was found that differences between patient-report and patient files data were amplified with increasing utilisation. This trend was also observed in a previous study conducted in a sample of community-dwelling men for a number of healthcare usage parameters including inpatient overnight stays and ambulatory physician visits [8].

If a recommendation had to be made on whether to use patient files or patient-report for the determination of number of visits, the latter would be a sensible option. Economic data are likely to be of greater use to policy-makers if they cover a broad spectrum of variables including lost productivity and out-of-pocket expenses, which are best determined using patient-report. In addition, since this method is less labour intensive for participating clinicians, it may lead to better participation rates and thus more representative results.

Despite these strengths, the economic significance of using patient-report over patient files for the determination of number of visits is unknown. In the present study, participants under-reported the number of visits by on average one visit when compared to the number of visits recorded in patient files. It is likely that the impact on overall cost calculations when varying the number of visits by one additional or less visit in an average total of visits may be minor. However, to confirm this in future studies on the costs of treatments, a sensitivity analysis should be used assessing the robustness of results to variations in the data on the number of visits made to the clinic as reported by patients.

The present study must be viewed in light of its limitations. Although patient files have previously been used as a reference comparator, it is uncertain whether this method represents the more reliable source of data on number of visits. Indeed, these data may have been omitted from files by mistake or recorded in such a form that they were not easily accessible to the clinic staff responsible for returning the number of patient visits made to the clinic during the study period. While the participants formed a representative sample of the study population, the findings of this study may not be generalisable to other populations, settings and designs. For instance, it is unclear whether longer recall periods would yield less accurate results.

## Conclusion

The present study showed that there were differences between the data on number of visits made to chiropractic clinics over a three-month period based on patient-report and actual visits recorded in patient files. Thus, if patient-reported data on number of visits are to be used in evaluations of costs of treatments, the impact of potential errors in reporting this information should be taken into account.
